# Glacial–interglacial Nd isotope variability of North Atlantic Deep Water modulated by North American ice sheet

**DOI:** 10.1038/s41467-019-13707-z

**Published:** 2019-12-18

**Authors:** Ning Zhao, Delia W. Oppo, Kuo-Fang Huang, Jacob N. W. Howe, Jerzy Blusztajn, Lloyd D. Keigwin

**Affiliations:** 1 Geology and Geophysics Department, Woods Hole Oceanographic Institution, Woods Hole, MA USA; 2 Climate Geochemistry Department, Max Planck Institute for Chemistry, Mainz, Germany; 3 Institute of Earth Sciences, Academia Sinica, Taipei, Taiwan

**Keywords:** Palaeoceanography, Marine chemistry, Geochemistry

## Abstract

The Nd isotope composition of seawater has been used to reconstruct past changes in the contribution of different water masses to the deep ocean. In the absence of contrary information, the Nd isotope compositions of endmember water masses are usually assumed constant during the Quaternary. Here we show that the Nd isotope composition of North Atlantic Deep Water (NADW), a major component of the global overturning ocean circulation, was significantly more radiogenic than modern during the Last Glacial Maximum (LGM), and shifted towards modern values during the deglaciation. We propose that weathering contributions of unradiogenic Nd modulated by the North American Ice Sheet dominated the evolution of the NADW Nd isotope endmember. If water mass mixing dominated the distribution of deep glacial Atlantic Nd isotopes, our results would imply a larger fraction of NADW in the deep Atlantic during the LGM and deglaciation than reconstructed with a constant northern endmember.

## Abstract (web summary)

The Nd isotope composition of seawater has been used to reconstruct past changes in the various contributions of different water masses to the deep ocean, with the isotope signatures of endmember water masses generally assumed to have been stable during the Quaternary. Here, the authors show that deep water produced in the North Atlantic had a significantly more radiogenic Nd signature during the Last Glacial Maximum compared to today.

## Introduction

The Atlantic meridional overturning circulation (AMOC) plays an important role in global climate change^1^. The overturning cell was argued to have been much shallower than today during the LGM [~24-18 thousand years (ka) before present (BP)], and during North Atlantic deglacial cold events, Heinrich Stadial 1 (HS1; ~17.5–14.6 ka BP) and the Younger Dryas (YD; ~12.9–11.7 ka BP)^2–4^. Due to the absence of biological fractionation effects and the quasi-conservative behaviour of dissolved Nd isotopes in the Atlantic basin that is dominated by strong advection^5^, the Nd isotope composition has been widely applied to study changes in water mass volumes and mixing in the past^2–8^. The underlying approach assumes a simple mixing scenario between two endmembers in the Atlantic whose isotope values have remained unchanged through time. The northern source water (NSW) has a modern mean *ε*_Nd_ (normalized ^143^Nd/^144^Nd ratio to the chondritic uniform reservoir^9^ in parts per 10,000) of about –13, reflecting the input of unradiogenic Nd from old continental rocks around the North Atlantic^10,11^. The southern source water [SSW, including Antarctic bottom water (AABW) and Antarctic intermediate water (AAIW)] is a mixture of Atlantic and Pacific (surrounded by rocks that contain more radiogenic Nd) waters and has an *ε*_Nd_ signature between –9 and –8 today^12^.

Data from ferromanganese crusts^13^ and deep-sea corals^14^ have been interpreted as indicating a relatively stable North Atlantic *ε*_Nd_ endmember through the Late Quaternary. However, the low temporal resolution of Nd isotope data (tens of thousands of years per sample) and high chronological uncertainty of ferromanganese crust samples^13^ and the poor temporal coverage of deep-sea coral samples, notably their absence during the LGM in the western North Atlantic^15^, raise questions about the inferred endmember stability, especially for the LGM interval. Previous studies have hypothesized that the North Atlantic *ε*_Nd_ endmember was more radiogenic during the LGM^16,17^, but so far there is no reliable record that demonstrates the glacial–interglacial evolution of this endmember.

Here, we present new evidence that the North Atlantic Nd isotope endmember was more radiogenic than modern at the LGM and became progressively less radiogenic during the last deglaciation. We suggest that this trend was caused by the retreat of the North American ice sheet (NAIS), which uncovered landmasses with unradiogenic Nd isotope signatures that weathered and resulted in the deposition of detritus from those regions into the Atlantic Ocean. Changes in deep water formation sites may have also contributed to the endmember change.

## Results

### A new *ε*_Nd_ record for the NSW

**Fig. 1:**
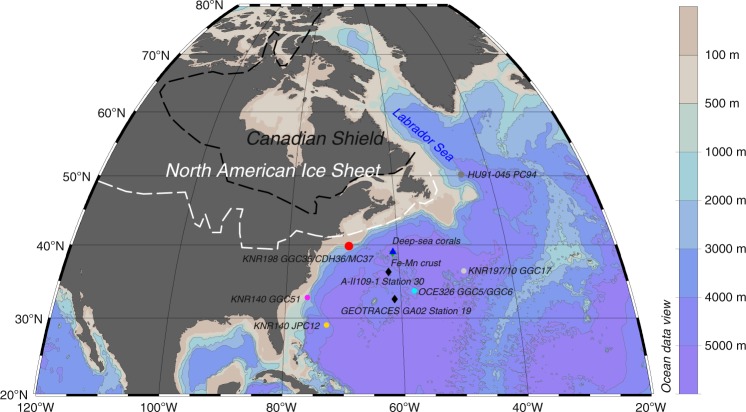
North Atlantic sites and bathymetry. Sediment cores (dots): KNR198-GGC35/CDH36/MC37 (40°N, 69°W, 1820 m; this study), KNR140-GGC51 (1790 m) and JPC12 (4250 m)^17,36^, OCE326-GGC5/GGC6 (4550 m)^2,32^, KNR197/10-GGC17 (5010 m)^49^ and HU91-045-PC94 (3450 m)^64^; Deep-sea corals (1180–1380 m; triangle)^33^; Ferromanganese crust BM1969.05 (1800 m; inverted triangle)^13^; Seawater samples (diamonds): A-II109-1 Station 30^10^ and GEOTRACES-GA02 Station 19^11^. Black and white dash lines delineate the boundary of the Canadian Shield and the maximum extent of the North American ice sheet during the LGM, respectively^34^. Figure made with Ocean Data View^71^.

We reconstruct the NSW *ε*_Nd_ evolution by measuring *ε*_Nd_ on post-depositional iron–manganese coatings of planktonic foraminifera, which has been shown to be a reliable recorder of seawater *ε*_Nd_ (ref. ^2^). Our reconstruction is based on a composite record obtained from three sediment cores with high sedimentation rates from the same location which together cover the interval since ~21 ka BP (see the “Methods” section). Located at ~1800 m on the New England Slope (Fig. 1), our site is within the range of depths having relatively high LGM stable carbon isotope values (*δ*^13^C > 1‰ for depths shallower than 2000 m in the North Atlantic)^18^, suggesting that it was bathed predominantly by glacial NSW. Records of ^231^Pa/^230^Th, a kinetic proxy for AMOC strength, from the North Atlantic also suggest a vigorous overturning circulation above ~2500 m during the LGM and the last deglaciation^19,20^. In addition, our site is located in the western subtropical North Atlantic, in the region where seawater stations were used to define the modern *ε*_Nd_ endmember^10,11^. Finally, the site is not significantly influenced by Nd isotope labelling by ice-rafted debris (IRD) as is the case in the subpolar region^21^.

**Fig. 2:**
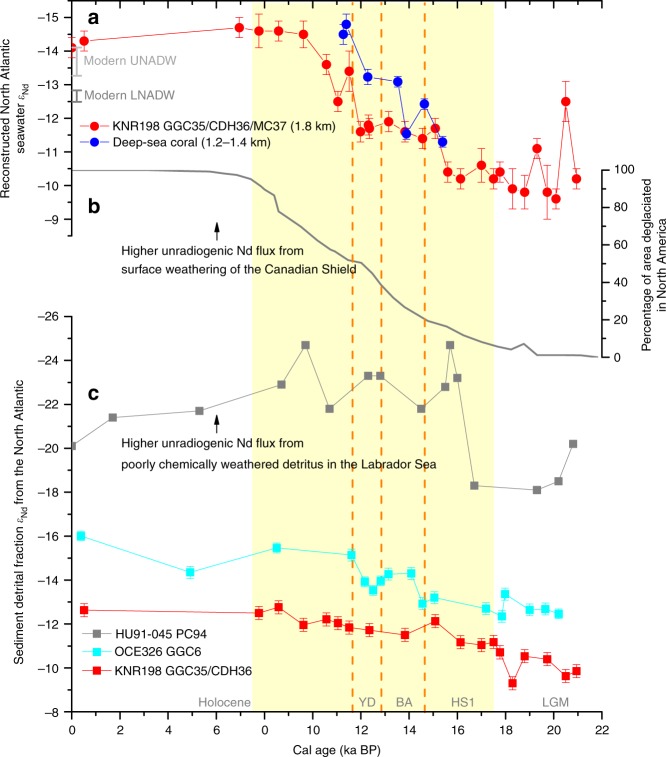
Reconstructed North Atlantic seawater *ε*_Nd_ in comparison with North American ice sheet deglacial history and North Atlantic detrital *ε*_Nd_ records. **a** *ε*_Nd_ of foraminiferal Fe–Mn coatings from this study compared to deep-sea coral records^33^ and modern *ε*_Nd_ ranges for Upper and Lower NADW based on GEOTRACES GA02 Station 19 (ref. ^11^). **b** Percentage of area deglaciated in North America with reference to the maximum NAIS extent during the LGM (ref. ^34^). **c** Sediment detrital fraction *ε*_Nd_ from HU91-045-PC94 (ref. ^64^), OCE326-GGC6 (ref. ^31,72^), and KNR198-GGC35/CDH36 (this study). Error bars plotted are 2*σ*. Yellow bar represents the interval of NAIS deglaciation, as also in later figures. Vertical orange lines denote boundaries between climate intervals. YD: Younger Dryas; BA: Bølling–Allerød; HS1: Heinrich Stadial 1; LGM: Last Glacial Maximum.

Today, several water masses from different sources contribute to NADW, but the resulting southward flowing NADW *ε*_Nd_ endmember at 30–40°N in the subtropical western North Atlantic^10,11^ is relatively homogeneous due to mixing and boundary exchange in the subpolar region^22^. As reported in a recent study, the mean *ε*_Nd_ values of the upper and lower NADW are within 1 *ε*-unit from each other in the subtropics, with the less radiogenic values in upper NADW reflecting a greater Labrador Sea Water component^11^ (Fig. 2a, Supplementary Fig. 1). By contrast the NSW–SSW gradient is >4 *ε*-units^12^.

The multi-core top *ε*_Nd_ of −14.1 ± 0.3 from our site agrees well with the seawater values at similar depths from stations near our site (A-II109-1 Station 30: −13.5 ± 0.4 and GEOTRACES GA02 Station 19: −13.54 ± 0.3; Fig. 1, Supplementary Fig. 1)^10,11^. Because of the strong and fast Deep Western Boundary Current, our site might record a higher percentage of NSW than the seawater stations. The *ε*_Nd_ of Labrador Sea Water (−14.15 ± 0.07)^5^, the main component of upper NADW, is in better agreement with the core-top value. Although we do not have detrital data for the multi-core top, the sediment detrital *ε*_Nd_ from the gravity-core top (−12.6 ± 0.3 at 0.5 ka BP) is significantly different from the foraminifera-derived *ε*_Nd_ (gravity-core top: −14.3 ± 0.3; multi-core top: −14.1 ± 0.3).

Our reconstructed NSW *ε*_Nd_ signatures during the LGM and deglaciation are significantly different from modern values (Fig. 2a). The samples older than 17.5 ka BP average −10.4 ± 1.0 (*N* = 8), about four *ε*-units more radiogenic than the core-top value. In addition, NSW *ε*_Nd_ was variable during the LGM, with some time intervals having less radiogenic Nd signatures (Fig. 2a). The *ε*_Nd_ decreases by more than 1 *ε*-unit in late HS1 and then remains relatively stable until the YD. After the YD, the *ε*_Nd_ exhibits a large decrease, reaching stable values (~−14.6) at ~10 ka that are maintained until at least 7 ka (Fig. 2a).

### Reliability of the downcore ε_Nd_ record

As mentioned above, detrital *ε*_Nd_ is less negative (more radiogenic) than authigenic *ε*_Nd_ at our site today. The detrital *ε*_Nd_ is relatively stable downcore, with a slight trend towards lower values since the LGM (Fig. 2c). By contrast, the downcore authigenic Nd isotope composition was much more radiogenic than today, leading to similar values of detrital and authigenic *ε*_Nd_ during the intervals of the deglaciation, when the authigenic record changed from being more radiogenic than the detrital record to less radiogenic than the detrital. This does not mean that the authigenic *ε*_Nd_ values during those intervals are modified by that of the detrital material. In fact, deglacial rare-earth element (REE) data show that our authigenic *ε*_Nd_ record is minimally influenced by diagenetic alteration after the authigenic *ε*_Nd_ is formed (Supplementary Fig. 2). Moreover, authigenic *ε*_Nd_ is usually insensitive to alteration after burial because of the high Nd concentration in that phase^23^. On the other hand, detrital sediments consist of components with distinct *ε*_Nd_ and reactivities, and thus could release Nd that is isotopically different from the bulk detrital digestion^23,24^. Therefore, a significant offset between authigenic and detrital *ε*_Nd_ values may be neither a sufficient nor a necessary condition for no influence of detritus.

**Fig. 3:**
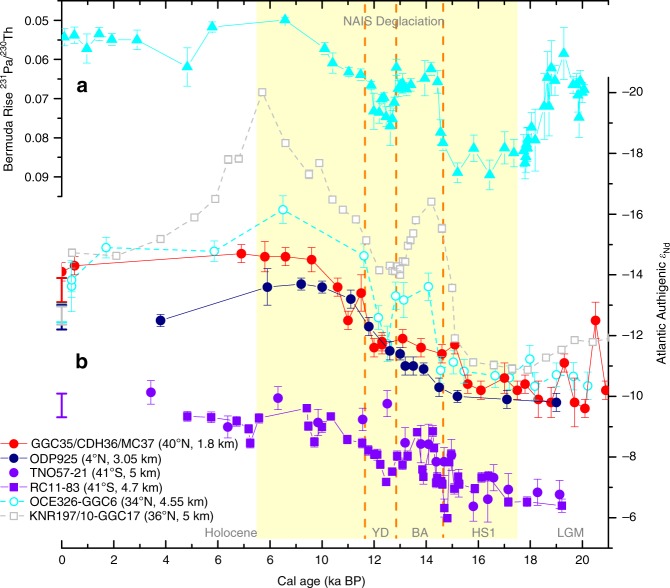
Atlantic authigenic *ε*_Nd_ records since the LGM. **a** ^231^Pa/^230^Th of OCE326-GGC5 from the Bermuda Rise^32^. **b** Authigenic *ε*_Nd_ records from the mid-depth (this study) and deep North Atlantic^2,49^, and the deep equatorial^31^ and South^4,73^ Atlantic. Core locations are plotted on a meridional transect of Atlantic salinity today in Supplementary Fig. 8. Foraminiferal and fish teeth data are shown by dots, while the squares represent data based on sediment leachates. Records that are probably influenced by a bottom source of unradiogenic Nd from poorly chemically weathered detritus^31^ are shown by open symbols connected with dashed lines. Seawater data from the water samples closest to each core are shown with bars at 0 ka BP (see Supplementary Data 1 for details). All error bars are 2*σ*. YD: Younger Dryas; BA: Bølling–Allerød; HS1: Heinrich Stadial 1; LGM: Last Glacial Maximum.

Although the evidence in hand suggests that the foraminifera-based authigenic record is minimally influenced by local detrital modification after burial, a significant influence of benthic fluxes before the formation of authigenic coatings must be ruled out. Benthic fluxes away from the water source regions could modify the deep water *ε*_Nd_ expected from endmember mixing^5^, as proposed for the deep (deeper than 3–4 km) Pacific where the residence time of water is long, and because there is a large seafloor area at those depths^25^. Several observations suggest that our authigenic *ε*_Nd_ record is minimally influenced by benthic fluxes. First, rapid *ε*_Nd_ variations in our authigenic record (e.g., during the LGM and the early Holocene) are not present in the detrital record, suggesting that the authigenic record reflects seawater rather than variations in the benthic fluxes near our site (Fig. 2). Second, the seafloor is generally deeper than 4 km in the subtropical North Atlantic (Fig. 1), so any benthic fluxes at our shallow site (1.8 km) would have been effectively diluted by mixing along the same isopycnal surface with waters that were unlikely to have experienced benthic fluxes. Finally, although deep advection was in general weak during stadials, especially HS1 (Fig. 3a), radiocarbon-based ventilation age estimates suggest that the water residence time shallower than ~2.5 km was still relatively short (Supplementary Fig. 3). A likely increase in the surface reservoir age of the deep water formation region during those intervals^26^ would suggest even shorter residence time.

Another important factor for benthic fluxes could be the chemical weathering maturity of the detritus. The detrital Nd isotope composition (Fig. 2c) suggests that the detritus at our site has been consistently dominated by sediments from the nearby coast^27^, which are relatively well chemically weathered, rather than those from the Canadian Shield, which are poorly chemically weathered due to strong glacial denudation^28^. The absence of unradiogenic shifts in the detrital *ε*_Nd_ during IRD peaks at our site indicates that the IRD (almost pure quartz) was brought by icebergs that calved also near the New England coast (e.g., the Gulf of Maine; Fig. 1) rather than from the Canadian Shield (Supplementary Fig. 4). Since chemical weathering flux is anti-correlated with substrate exposure time^28^, well chemically weathered detritus (like sources near our site) would result in less significant benthic fluxes into the bottom water. In contrast, detritus derived from relatively poorly chemically weathered rocks, such as the volcanic rocks around the Pacific^25^ and the newly exposed Canadian Shield^28^, likely releases larger benthic fluxes. Poorly chemically weathered sediments entering into the abyssal ocean near the Labrador Sea when the NAIS retreated rapidly, e.g., during warm intervals like the Bølling–Allerød (BA) and the early Holocene^29^, could have led to large benthic fluxes and thus explain the very unradiogenic Nd signatures reported from the Corner Rise in the deep northwestern Atlantic (KNR197/10-GGC17: 5 km, 36°N; Figs. 1 and 3b)^30^. The moderately negative *ε*_Nd_ excursions during the early Holocene^2^ and interstadials^3^ at the Bermuda Rise (4.55 km, 34°N), which is shallower and farther away from the depocenter than the Corner Rise, could have been related to this process as well^31^. The detrital influences seem larger but the water residence time was shorter in the deep Atlantic during interstadials than stadials (Fig. 3)^3,32^, suggesting that the amount of poorly chemically weathered detritus has been more important than water residence time for the impact of benthic fluxes.

**Fig. 4:**
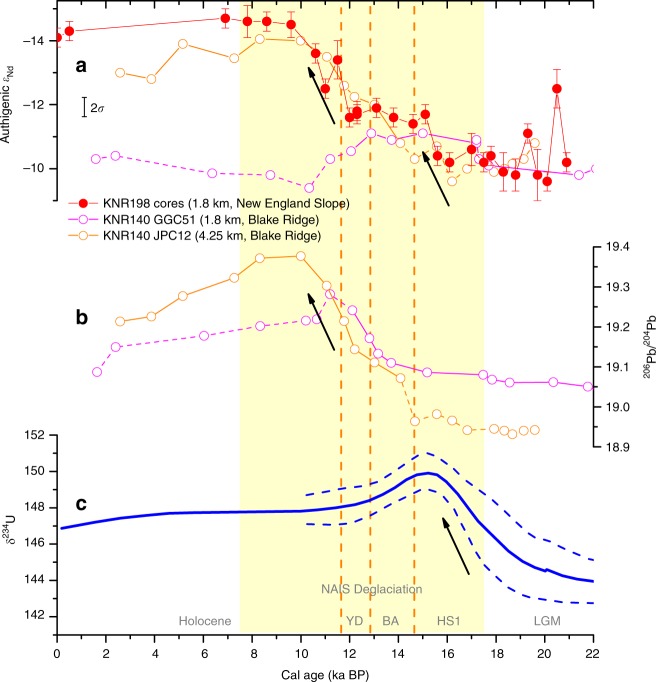
Reconstructed isotope composition variability of Nd, Pb and U in seawater from the North Atlantic. **a** Authigenic *ε*_Nd_ based on foraminifera from our study (with 2*σ* error) and sediment leachates from two Blake Ridge cores (with their 2*σ* errors represented by the black error bar)^17^. **b** Authigenic ^206^Pb/^204^Pb based on sediment leachates from the two Blake Ridge cores^36^, with error bars smaller than the symbols. For **a** and **b**, the data in each core for the interval that are most likely influenced by sediment redistribution^17^ are connected by a dashed line. **c** Reconstructed δ^234^U of the upper (0.7–2.1 km) tropical North Atlantic, with the mean value shown as a solid line and 2*σ* error as dashed lines^45^. YD: Younger Dryas; BA: Bølling–Allerød; HS1: Heinrich Stadial 1; LGM: Last Glacial Maximum. Black arrows in this figure denote potential influences of weathering fluxes related to NAIS deglaciation: the increased subglacial melting and detritus discharge during HS1 and the enhanced chemical weathering of the Canadian Shield in the early Holocene.

Our *ε*_Nd_ endmember estimates for the LGM and the early deglaciation are similar to those from 1790 m on the Blake Ridge (KNR140-GGC51; Figs. 1 and 4a)^17^, which were based on sediment leachates. However, our values diverge significantly from the late deglacial and Holocene values of this Blake Ridge record, which was compromised by downslope and lateral sediment redistributions^17^ that resulted in significantly more radiogenic signatures (Fig. 4a). Instead, another record from the Blake Ridge from 4250 m agrees more closely with our record for the Holocene and the late deglaciation, times when it was less likely influenced by sediment redistribution^17^ (Fig. 4a). Deep-sea corals at 1180–1380 m from New England Seamounts provide a relatively continuous *ε*_Nd_ record during ~15.5–11 ka BP (Fig. 2a)^33^. The coral data contain a similar trend as our record but show generally more negative values (unradiogenic Nd), consistent with the seawater *ε*_Nd_ difference today between their depths (Supplementary Fig. 1). It has been suggested that during the deglaciation, the deep-sea coral record may have been influenced by vertical movement of thermocline^33^, which has more positive *ε*_Nd_. However, the influence of thermocline variability on our sediment record from 1.8 km must have been minimal, and was also likely limited on the coral record because otherwise it would have more positive *ε*_Nd_ than our record, which is opposite of the observations (Fig. 2a). We also note that deep-sea corals probably record decadal-scale variations while our record should be viewed as a long-term smoothing of such signals during this interval (our record resolves rapid changes during the LGM due to the much higher sedimentation rate then; Fig. 2a). For instance, deep-sea corals from 1.7 to 2.6 km depth suggest high-amplitude *ε*_Nd_ variability (pulses reaching −14 from a background of about −11) around 15.4 ka BP^33^, which may indicate pulses of water with unradiogenic Nd (e.g., due to rapid changes in the location of deep water formation as discussed below).

### Mechanisms for the glacial–interglacial NSW ε_Nd_ evolution

Multiple processes likely have contributed to the evolution of the North Atlantic *ε*_Nd_ endmember since the LGM. First, the deglaciation of the NAIS exposed the Canadian Shield^34^, which is a major source of unradiogenic Nd for the North Atlantic Ocean^6^. The unradiogenic Nd isotope signature from riverine input was carried to the wider high-latitude North Atlantic by ocean currents as observed today^11^, and the Nd isotope signature of the surface ocean was then transported to the deep ocean by open-ocean deep convection^22,35^. Deglaciation of the Canadian Shield significantly increased the chemical weathering and transport of unradiogenic Nd into the high-latitude North Atlantic by continental runoff. Basal meltwater near the ice sheet edge, especially along the Labrador Sea coast (Fig. 1), likely also contributed. Since the NAIS extended beyond the Canadian Shield during the LGM and the Shield was likely not deglaciated until the YD^34^, riverine input likely contributed most to the *ε*_Nd_ evolution during the second half of the deglaciation (since the YD), which is also the largest change during the deglacial transition. This agrees very well with the published authigenic Pb isotope data from the western North Atlantic (Fig. 4), which recorded the increased weathering flux of radiogenic Pb from the freshly exposed continent during the early Holocene^36^.

Changes in the location of deep water formation likely also contributed to the *ε*_Nd_ endmember evolution, especially the rapid variations. The water mass bathing our site today is formed in the Labrador Sea and other regions like the Irminger Sea (the latter also called Labrador Sea Water)^37^. Based on modern conditions (Supplementary Fig. 5)^5^, a shift of water source to the Nordic Seas could explain the more positive glacial and deglacial *ε*_Nd_ in our record (with boundary exchange in the subpolar region not considered). However, this is not consistent with observations that suggest significantly weakened deep water formation^38^ and less radiogenic Nd signatures in the deep Nordic Seas during cold intervals^39^. In addition, our *ε*_Nd_ record shows a generally monotonic shift during the deglaciation, while the deep water formation strength in the Nordic Seas appears to have switched back and forth between strong and weak modes^38^. We thus believe that the formation site of the water mass bathing our site was still to the south of Iceland during the LGM and deglaciation, consistent with previous inferences that glacial North Atlantic intermediate and deep waters were formed in the subpolar region (Supplementary Fig. 5)^40–42^. Migration of the water formation site in the subpolar region might have contributed to some fraction of the glacial–interglacial offset of NSW *ε*_Nd_. However, because the surface *ε*_Nd_ is quite homogeneous in that region, probably as a result of the rapid mixing by the subpolar gyre^11^, a glacial *ε*_Nd_ endmember shift to ~−10 is hard to justify without a background change in the surface *ε*_Nd_ of the high-latitude North Atlantic (a result of changes in continental weathering as discussed above) (Supplementary Fig. 5). On the other hand, surface water *ε*_Nd_ along the western boundary of the subpolar gyre is less radiogenic (Supplementary Fig. 5). A shift of water formation site towards that boundary, where the surface is fresher but colder and may be suitable for deep water and brine formation, could have led to negative shifts in deep water *ε*_Nd_, such as the fluctuations during the LGM in our record (Fig. 2) and the pulses of unradiogenic Nd in deep sea corals during late HS1^33^. These shifts are quite rapid, and may imply changes in deep-water formation location and strength that occurred abruptly in response to atmospheric forcing^37^.

The Nd isotope composition of detritus in the subpolar North Atlantic might have also contributed to changes in NSW *ε*_Nd_ endmember. Like today^22^, if dense NSW formed in the Nordic Seas during some intervals in the past (e.g., the BA^38^), this NSW would acquire a Nd isotope signature similar to the upper NSW by boundary exchange in the western subpolar North Atlantic^11^, in addition to the entrainment and mixing between them^11,43^. During the glacial interval, the discharge of unradiogenic detritus for boundary exchange was probably reduced, and this reduction would enhance the background *ε*_Nd_ change in the surface ocean by pushing the NSW endmember towards more radiogenic values (Fig. 2). In the early deglaciation, discharge of sediments frozen within the ice sheet base probably increased together with the ice discharge rate^44^. This could have led to the less radiogenic detritus near the Labrador Sea (Fig. 2c), which might have influenced NSW *ε*_Nd_ by boundary exchange afterwards, as well as the reported release of excess ^234^U from subglacial melting to the Atlantic^45^ (Fig. 4). Although the fraction of sediments from the Canadian Shield has always been small at our site, the trend of our detrital Nd isotope signature towards slightly less radiogenic values (in parallel with that from the Bermuda Rise) suggests that the average detrital Nd isotope composition in the subpolar region became less radiogenic during the deglaciation (Fig. 2c). The detrital materials were transported southward by deep currents^46^, with the influence of flow speed possibly also superimposed on the records.

### Implications and complications

**Fig. 5:**
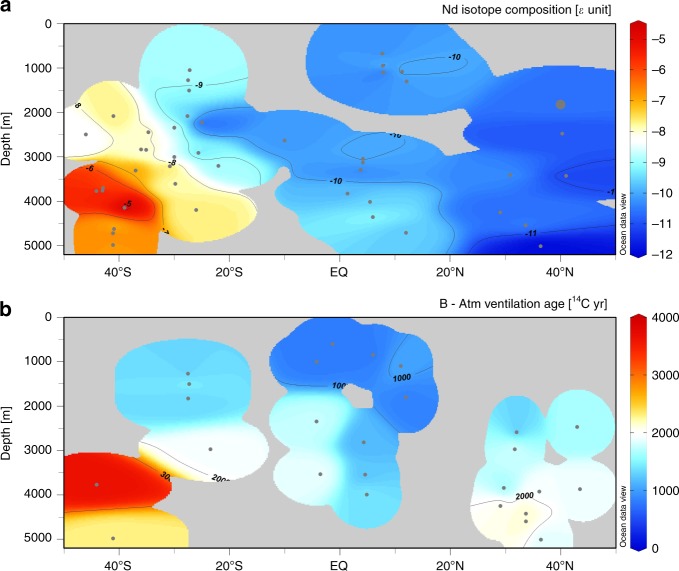
Compilation of *ε*_Nd_ and ventilation age data during the LGM (23–19 ka BP) from the western basin of the Atlantic. **a** Compilation of authigenic *ε*_Nd_. **b** Compilation of ^14^C age differences between deep ocean samples and the contemporaneous atmosphere. Data locations are shown by dots, with the large one at 40°N in **a** representing our cores. See the Compilation of *ε*_Nd_ and ^14^C data section in “Methods” section for details.

If variations in the Nd isotope signature of deep waters are dominated only by water mass fractions on the deglacial time scale, as is often assumed, our results imply a significantly larger influence of NSW during the LGM and the deglaciation than previously inferred from published deep Atlantic *ε*_Nd_ records (e.g., refs. ^2–4,7,47–49^). A recent study assuming quasi-conservative Nd isotope behaviour^7^ suggests that the fraction of NSW was >50% in the deep (>2500 m) North Atlantic during the LGM. Consistent with that study^7^, our results showing a radiogenic shift by about four *ε*-units in the northern endmember would imply almost pure NSW bathing the deep North Atlantic as far south as the northern subtropics (Fig. 5a). In addition, with our new endmember constraint, the *ε*_Nd_ value of the deep equatorial Atlantic was much closer to the North Atlantic than the deep South Atlantic (Fig. 5a), suggesting that NSW was still volumetrically important in the deep Atlantic basin during the LGM. The inference from Nd isotopes is generally consistent with our compilation of radiocarbon-based ventilation age estimates, which suggest younger ventilation age in the deep North Atlantic than the deep Southern Ocean (Fig. 5b). Note, however, that planktonic–benthic radiocarbon age differences are generally as large in the North Atlantic as the Southern Ocean (Supplementary Fig. 6).

If the core of NSW export was shallower during the LGM, as inferred from sedimentary ^231^Pa/^230^Th (refs. ^19,20^) and stable carbon isotopes (*δ*^13^C)^50^, then a significant NSW fraction in the deep Atlantic basin could only be maintained if AABW flow to the deep Atlantic weakened significantly, perhaps due to more equatorward southern westerly winds during the LGM^51^. The compilation of *ε*_Nd_ suggests that the deep Atlantic sector of the Southern Ocean was strongly stratified, implying a very dense AABW that was isolated from the overlying Circumpolar Deep Water during the LGM (Fig. 5a), similar to the Pacific sector^52^. Our results showing similar values in the shallow tropical Atlantic and at our North Atlantic core site also suggest a significantly weaker AAIW intrusion during the LGM (Supplementary Fig. 7), consistent with previous studies^16,53^.

Taking the small gradient between our North Atlantic record and a foraminifera-based authigenic *ε*_Nd_ record from the deep equatorial Atlantic (ODP925: 3.05 km) at face value would imply a much larger fraction of NSW than previously estimated in the deep equatorial Atlantic throughout the deglaciation^31,49^ (Fig. 3b). In addition, our radiocarbon-based ventilation age estimates suggest that the deep western Atlantic was as well ventilated during HS1 as the LGM (Supplementary Fig. 3). These findings contradict many previous studies based on *ε*_Nd_ and ^231^Pa/^230^Th suggesting that SSW dominated the whole deep Atlantic with the AMOC in a collapsed or significantly weakened state during HS1 (e.g., refs. ^2,3^) and other Heinrich events near glacial maxima^3^, but is consistent with a small number of studies suggesting a strong presence of North Atlantic source waters^54,55^.

The inference above for the water mass geometry of the glacial deep Atlantic is, however, not consistent with previous studies based on *δ*^13^C. It is widely known that the *δ*^13^C below ~2.5 km in the North Atlantic was significantly lower during the LGM than today^18^. A model simulation that fits existing *δ*^13^C (and Cd) data suggests that the NSW fraction in the deep North Atlantic decreased from 60% to 90% in the modern to about 50% during the LGM^50^, and an updated study suggests an even greater reduction, with the glacial boundary between NSW and AABW (as represented by the 50% to 50% isopleth) shoaling to above 4 km^53^. The inconsistency between *ε*_Nd_, ^14^C, and *δ*^13^C must originate from differences associated with each tracer. With a weaker advection in the deep Atlantic than today, non-conservative processes (e.g., benthic fluxes and reversible scavenging)^5^ were likely more significant in modifying Nd isotope composition derived from water mass mixing in the glacial deep Atlantic, especially for abyssal depths (>4 km). Those processes are difficult to quantify, so estimates of water mass fractions in the past based on *ε*_Nd_ and associated errors are uncertain. Radiocarbon ventilation age estimates that are not based on independent chronologies are also uncertain, as the applied surface reservoir age has a large influence on the benthic minus atmospheric ventilation age^56^ (Supplementary Fig. 6). In addition, changes in the surface reservoir age at high latitudes could mask the deep ventilation (both benthic–atmospheric and benthic–planktonic) ages by modifying the preformed age of waters that supply the deep ocean^57^. As for *δ*^13^C, variations in endmember values could have a large influence on deep water mass fraction estimates, but are still uncertain (e.g., ref. ^58^). A NSW source from brine formation might also influence deep water *δ*^13^C in some locations^59^. Moreover, *δ*^13^C may have been reduced in the glacial deep North Atlantic due to greater accumulation of respired carbon that results from weaker ventilation^7,50^. Future studies focusing on reconciling the discordant results in the abyssal North Atlantic will help shed light on the glacial deep Atlantic water mass mixture.

We further note that there are complications for interpreting the glacial and deglacial *ε*_Nd_ records. First, in addition to *ε*_Nd_, the Nd concentration of seawater in the past has probably also changed. For example, given the likely decreased fluxes of Nd resulting from both lower riverine input and reduced deposition of poorly chemically weathered detritus in the subpolar region, the glacial Nd concentration of the NSW could be lower than today (Fig. 2b and c). On the other hand, a likely longer residence time of waters in the deep Atlantic (Fig. 5b) would increase the contact time of NSW with the detritus in the subpolar region, and thus tend to raise the glacial Nd concentration of NSW. Uncertain Nd concentrations remain a factor preventing accurate estimates of water mass fractions. Another potential complication for interpreting the glacial and deglacial *ε*_Nd_ records is the *ε*_Nd_ gradient between NSW and SSW. Our record suggests that the glacial NSW *ε*_Nd_ was about four *ε*-units more positive than today. Because SSW is a mixture of Atlantic and Pacific waters^12^, if we assume the Pacific endmember and the mixing ratio are stable, the SSW *ε*_Nd_ would also be more positive but with a smaller amplitude relative to NSW. On the other hand, a weaker deep Atlantic advection in the past could lead to a smaller North Atlantic contribution to SSW, thus pushing the SSW endmember towards the Pacific, a more positive value. Again, this reasoning is qualitative given the uncertainties in endmember Nd concentrations. Regardless, the NSW–SSW gradient appears to have been smaller than today, as suggested by the reduced glacial and deglacial offset between the deep South Atlantic records and our record (Fig. 3b). A smaller NSW–SSW gradient of *ε*_Nd_ would lead to a greater uncertainty in the reconstructions of water mass fractions, and could also lead to a greater relative influence of non-conservative processes in influencing Nd isotope records. Thus, while our results suggesting a more radiogenic glacial NSW endmember would imply a larger fraction of NSW in the glacial and deglacial deep Atlantic than assuming a constant NSW endmember, there remain uncertainties that need to be resolved in future studies.

Superimposed on potential modulations on longer timescales^60^, the growth and decay of the NAIS has probably regulated the weathering and input of unradiogenic Nd into the ocean and thus the evolution of the North Atlantic Nd isotope composition on glacial–interglacial timescales. Similar temporal patterns in other isotope systems support common drivers that are most likely related to changes in ice sheet-modulated weathering, e.g., continental runoff^36^ for Pb and Nd and subglacial discharge^45^ for U and Nd (Fig. 4). Our study contributes to a growing body of evidence that chemical weathering modulated by the waxing and waning of ice sheets had an important influence on the isotope composition of multiple elements (e.g., U^45^, Pb^36,61^, Hf^62^) in Atlantic seawater during the Quaternary.

## Methods

### Cores and chronology

Gravity core GGC35, piston core CDH36 and multi-core MC37 were taken from the New England Slope at 1820 m during research cruise KNR198 in 2010. The age model of GGC35/CDH36 was derived from radiocarbon dates determined in National Ocean Science Accelerator Mass Spectrometry facility at Woods Hole Oceanographic Institution (WHOI), calibrated with Marine 13 (ref. ^63^) and no extra reservoir age correction (Supplementary Data 1). Apparent outliers are not included in the age model. Chronologies that are based on radiocarbon dating from cited records were recalibrated with IntCal13/Marine 13 (ref. ^63^) with reservoir ages taken from the original publications, except that radiocarbon dates from HU91-045 PC94 (ref. ^64^) are calibrated with new reservoir age estimates of the high-latitude North Atlantic^26^. Updated correlation-based chronologies^65^ are used for TNO57-21 and RC11-83. Age models of KNR140 JPC12 and GGC51 have been updated with new ^14^C dates (see Supplementary Data 1 for details). The main changes for JPC12 are within the LGM-HS1, with the updated ages now being younger than the previous age model^62^ by up to ~1400 yr. Locations of the cores discussed in this study are plotted on a meridional salinity transect of the western Atlantic in Supplementary Fig. 8.

### Nd isotope analysis

Sample preparation and Nd isotope analysis follow refs. ^48,66^. In brief, for each sample, ~2–3 mg of clean planktonic foraminifera were picked from the >150 μm size fraction. All foraminiferal chambers were crushed open to remove dirty particles and then were ultrasonicated with MilliQ-water and methanol to remove clay. Samples were dissolved in dilute acetic acid to minimize the risk of leaching any remaining detrital materials^2^. For detrital fractions, sediments from <63 μm size fraction were decarbonated and then leached with 1 M hydroxylamine hydrochloride solution in 25% acetic acid to remove Fe–Mn oxides. Then the residues were digested in 4:1 hydrofluoric acid and perchloric acid. All dissolved samples were purified for Nd using a two-step (TRU spec + Ln resin, Eichrom Technologies) chromatography method^66^.

The Nd isotopic compositions were measured by Neptune MC-ICP-MS at WHOI and Neptune *PLUS* MC-ICP-MS at Institute of Earth Sciences, Academia Sinica. The standard exponential law was applied for correcting instrumental mass discrimination with normalization of ^146^Nd/^144^Nd to 0.7219. A JNdi-1 solution was used as a bracketing standard to monitor and correct for the instrumental mass fractionation. The ^143^Nd/^144^Nd ratios are reported as *ε*_Nd_, i.e., deviations from the chondritic uniform reservoir (CHUR: 0.512638)^9^:

1 $${\varepsilon}_{{\mathrm{Nd}}} = \left[ {\frac{{\left( {\frac{{{\mathrm{143}}_{{\mathrm{Nd}}}}}{{144_{{\mathrm{Nd}}}}}} \right)_{{\mathrm{Sample}}}}}{{\left( {\frac{{{\mathrm{143}}_{{\mathrm{Nd}}}}}{{{\mathrm{144}}_{{\mathrm{Nd}}}}}} \right)_{{\mathrm{CHUR}}}}}} \right] \times 10,000$$

Errors reported (2*σ*) are the external reproducibility determined from long-term runs of JNdi-1 standard, unless the internal error is larger than the external one, in which case a combined error $\left( {\sqrt {\left( {{\mathrm{internal}}\,{\mathrm{error}}^2 + {\mathrm{external}}\,{\mathrm{error}}^2} \right)} } \right)$ is used. Detailed data are presented in Supplementary Data 1.

### REE analysis

REE concentrations were measured on Element XR HR-ICP-MS (Thermo-Fisher) at Institute of Earth Sciences, Academia Sinica. By coupling with a high-sensitivity desolvation system Aridus II (Cetac), the formations of oxide/hydride were greatly reduced. Analyses were performed by measuring five calibration standards, which spanned the range of typical foraminiferal concentrations, to generate a linear calibration curve for each element. Samples were then run in blocks of five bracketed by two additional standards with typical foraminiferal REE concentrations as consistency check. These additional standards were further used to monitor and calculate the external reproducibility for each element, which was typically better than 3% (RSD). Intensities were corrected for instrumental drift during the analytical courses, and for oxide/hydride interferences. REE concentrations were then normalized to Post Archean Australian Shale (PAAS)^67^ (Supplementary Data 1).

### Compilation of ε_Nd_ and ^14^C data

Deep-sea authigenic *ε*_Nd_ data from the western Atlantic Ocean (locations unaffected by the sills of the eastern Atlantic as in ref. ^18^) are compiled for the LGM (a conservative time interval of 23–19 ka BP is used to minimize the influence of chronological uncertainties near the boundaries of the LGM), which is updated from a previous study^7^. Records north of 45°N were excluded due to potential labelling of IRD and volcanic ash^21^. The *ε*_Nd_ compilation consists of data based on foraminifera tests, fish debris and sediment leachates that are from sites where core-top values match seawater nearby. See Supplementary Data 1 for the compiled data.

Collection of published ^14^C data from the western Atlantic Ocean for the LGM (23–19 ka BP) and HS1 (17–15 ka BP; to minimize the influence of chronological uncertainties near the boundaries) is updated from a recent compilation^57^. The most significant difference between the compilation here and previously published compilations^57,68^ is that values based on miliolid species of benthic foraminifera are excluded due to generally much older ages than other species from the same sample^69^. For records with paired surface (planktonic foraminifera) ^14^C data available, benthic minus atmosphere (B−Atm) ventilation age was simply the sum of benthic−planktonic (B−P) ^14^C age difference and the inferred or assumed surface reservoir age from the original publication. Since surface reservoir age in some studies have been adjusted for the influence of lower atmospheric CO_2_ concentration during the LGM (~250 yr)^70^, in order to be consistent, 250 (150) yr is added to other LGM (HS1) records that use a modern reservoir age. For data with no paired surface ^14^C dates, e.g., those based on deep-sea corals, the ventilation age is the difference between the ^14^C age of the ocean and that of the contemporaneous atmosphere determined from the calendar age reported in the original paper and IntCal13 calibration curve^63^. Detailed information is shown in Supplementary Data 1.

## Supplementary information

Supplementary Information

Description of Additional Supplementary Files

Supplementary Data 1

## Data Availability

Data generated or compiled in this study are provided in Supplementary Data 1.
