# Publisher Correction: Glacial–interglacial Nd isotope variability of North Atlantic Deep Water modulated by North American ice sheet

**DOI:** 10.1038/s41467-020-17208-2

**Published:** 2020-07-01

**Authors:** Ning Zhao, Delia W. Oppo, Kuo-Fang Huang, Jacob N. W. Howe, Jerzy Blusztajn, Lloyd D. Keigwin

**Affiliations:** 1 Geology and Geophysics Department, Woods Hole Oceanographic Institution, Woods Hole, MA USA; 2 Climate Geochemistry Department, Max Planck Institute for Chemistry, Mainz, Germany; 3 Institute of Earth Sciences, Academia Sinica, Taipei, Taiwan

**Keywords:** Palaeoceanography, Marine chemistry, Geochemistry

Correction to: *Nature Communications* 10.1038/s41467-019-13707-z, published online 18 December 2019.

Equation (1) in the original version of this Article contained an error. This has now been corrected in the PDF and HTML versions of the Article.

