# Supplementary material for: Glacial–interglacial Nd isotope variability of North Atlantic Deep Water modulated by North American ice sheet
Source: Nat Commun. 2019 Dec 18;10:5773. doi: 10.1038/s41467-019-13707-z (PMC6920363; doi:10.1038/s41467-019-13707-z)
Supplement: Supplementary file 1 — Supplementary Information [file 41467_2019_13707_MOESM1_ESM.pdf]

Supplementary Information

Glacial-interglacial Nd isotope variability of North Atlantic Deep Water modulated by North American Ice Sheet

Zhao et al.

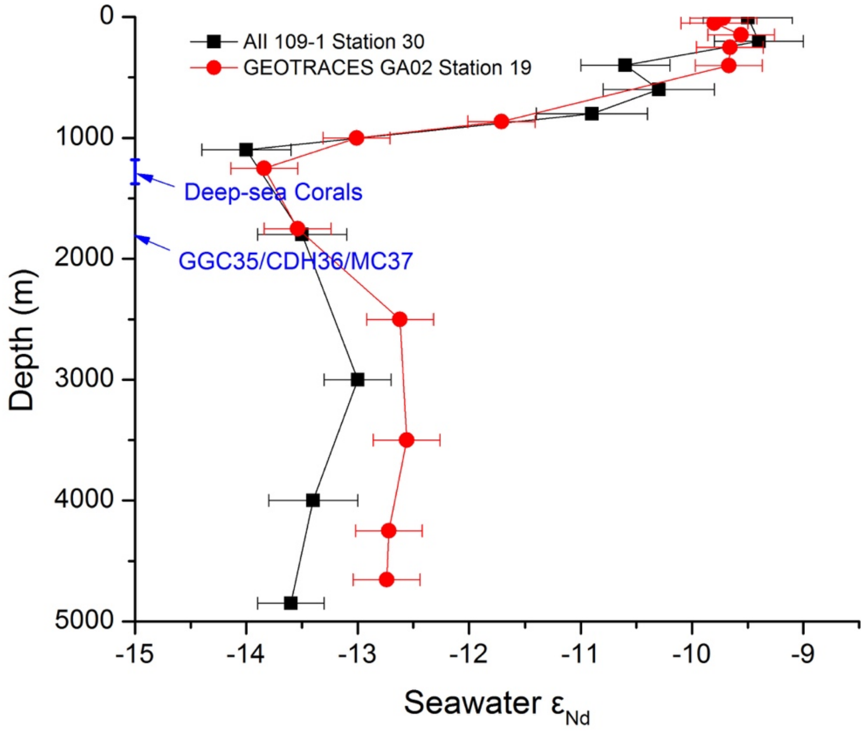

Supplementary Figure 1.

Vertical profiles of seawater ε_{Nd} in the western subtropical North Atlantic. The two stations are the ones closest to our site from two studies respectively: AII 109-1 Station 30 (ref. [1]) and GEOTRACES GA02 Station 19 (ref. [2]). Errors bars represent external reproducibility (2σ) for GEOTRACES GA02 Station 19, and 2σ of the measured mean for AII 109-1 Station 30 as the external errors are not reported. The depths of our cores and the deep-sea coral samples in Fig. 2 are noted.

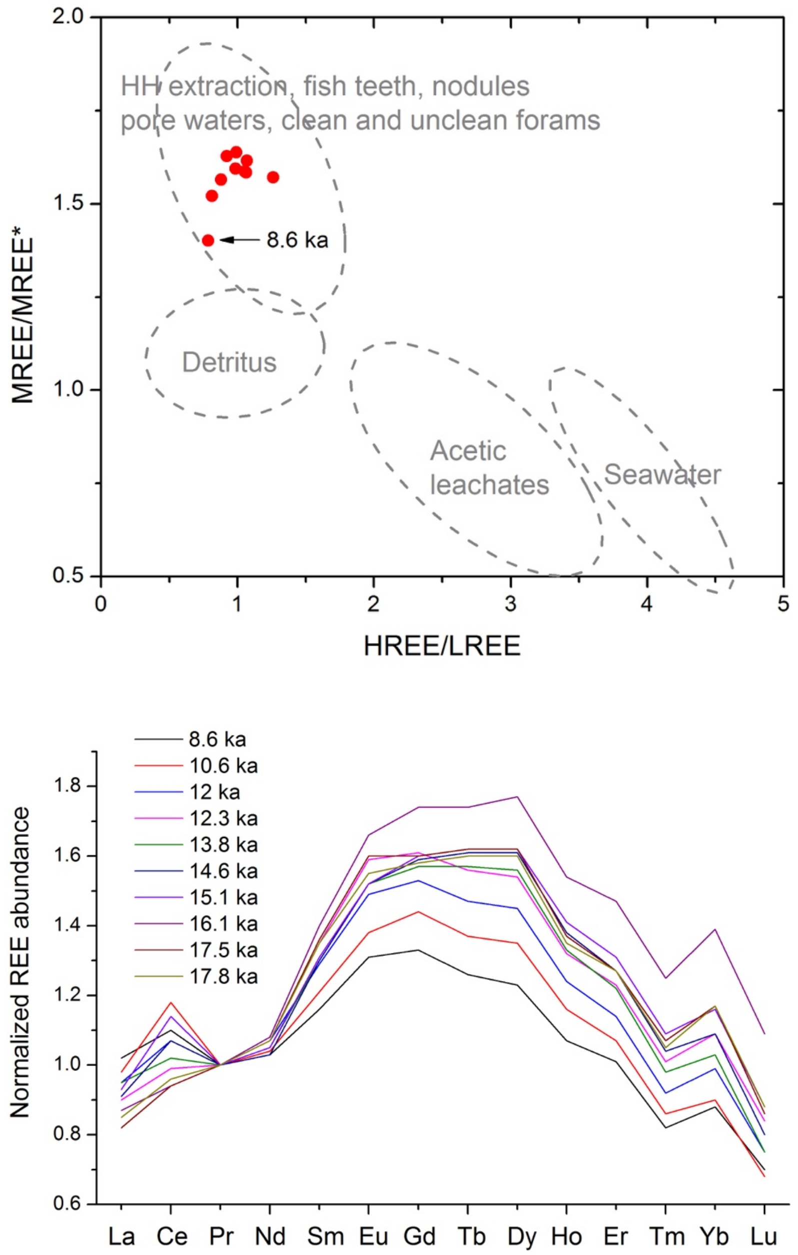

Supplementary Figure 2.

Upper panel: rare earth element ratios of foraminiferal Fe-Mn coatings from our study (red dots) plotted with values of various substrates defined in other studies (ref. [3] and references therein). LREE = La + Pr + Nd; MREE = Gd + Tb + Dy; HREE = Tm + Yb + Lu; MREE* = (LREE + HREE)/2. All element concentrations are normalized to Post Archean Australian Shale (PAAS; values taken from ref. [4]). The sample closest to the "detritus" region is from 8.6 ka, when the authigenic ε_{Nd} are clearly different from the detrital ε_{Nd} (Fig. 2). Lower panel: PAAS- and Pr-normalized REE patterns of the foraminiferal authigenic coatings. The clear MREE bulges are typical in authigenic coatings, but not in detrital materials. Although the interpretation of Ce anomaly is complicated, the lack of a negative Ce anomaly as seen in our record is also observed in other records from the western North Atlantic[5], which could be related to the young deep water in this region[6].

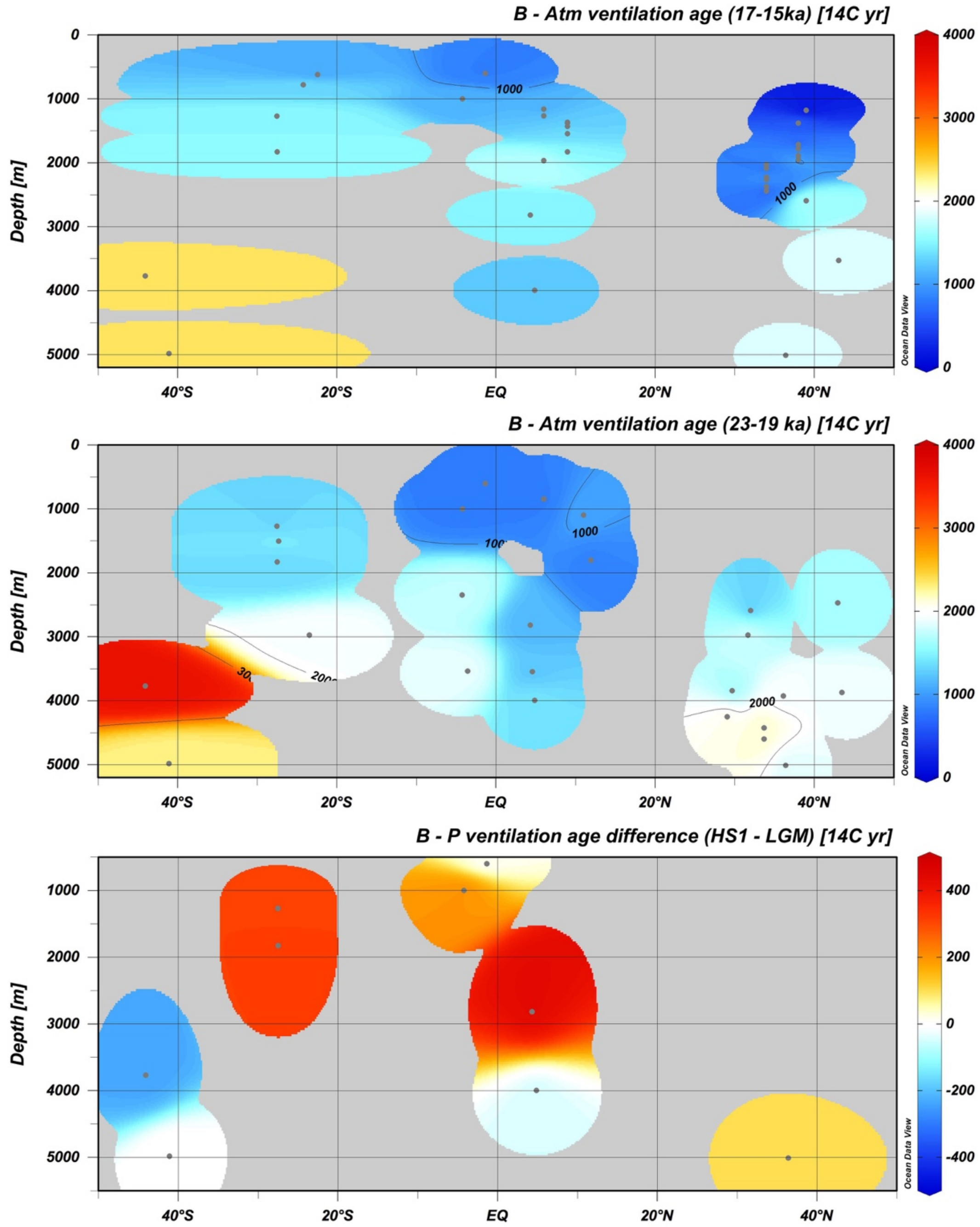

Supplementary Figure 3.

Compilation of ventilation age (benthic minus atmospheric ^{14}C age) from the western Atlantic basin for HS1 (17-15 ka BP; upper panel) in comparison with the LGM (23-19 ka BP; middle panel). Most of the data from 17-15 ka are based on deep-sea corals. Sites with records having data for both the LGM and HS1 are foraminifera-based, with the HS1-LGM differences of the benthic minus planktonic ventilation ages shown in the lower panel. The differences between the two intervals are generally small (note the different colour bar scale), especially when considering the data uncertainties and the possibility of a larger surface reservoir age in the NSW formation regions during HS1 (e.g., ref. [7]). See Methods and Supplementary Data 1 for the details of the compilation.

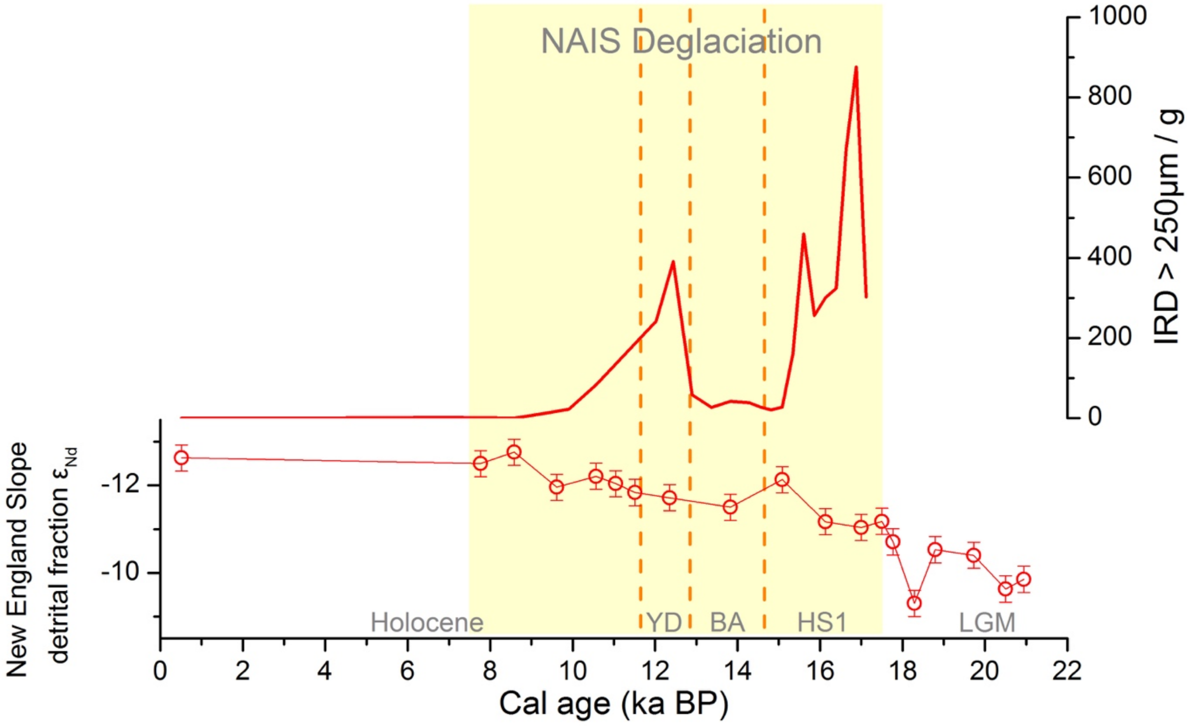

Supplementary Figure 4.

Upper panel: the abundance of IRD larger than 250 μm from KNR198 GGC35. IRD grains cover various sizes, with the grains larger than 63 μm being almost pure quartz at our site. Lower panel: detrital fraction ε_{Nd} from KNR198 GGC35/CDH36 with 2σ error bars.

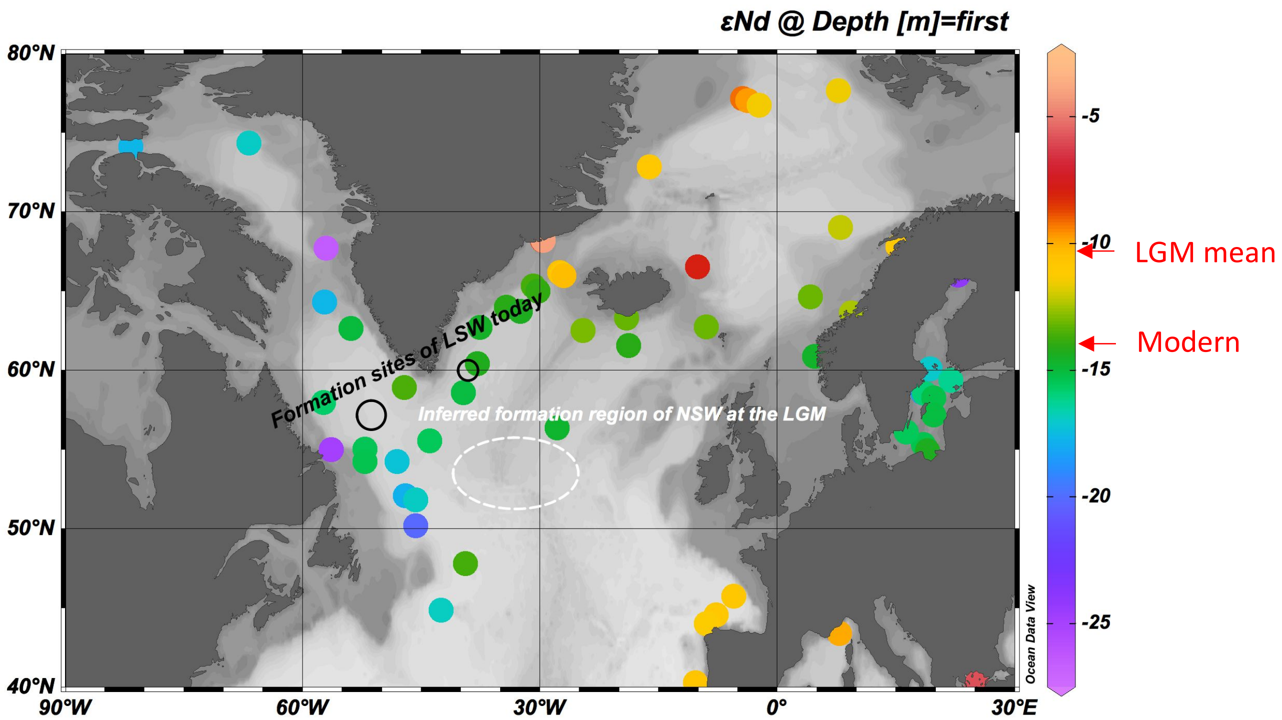

Supplementary Figure 5.

Surface ε_{Nd} of the northern North Atlantic. Data are compiled by van de Flierdt et al.[8]. The shallowest data (max depth: 50 m) from each station are plotted. The observed formation sites of the Labrador Sea Water today[9,10] and the inferred formation region for the North Atlantic intermediate and deep waters during the LGM[11] are noted. The modern and LGM mean ε_{Nd} of our record are marked to the right of the color bar.

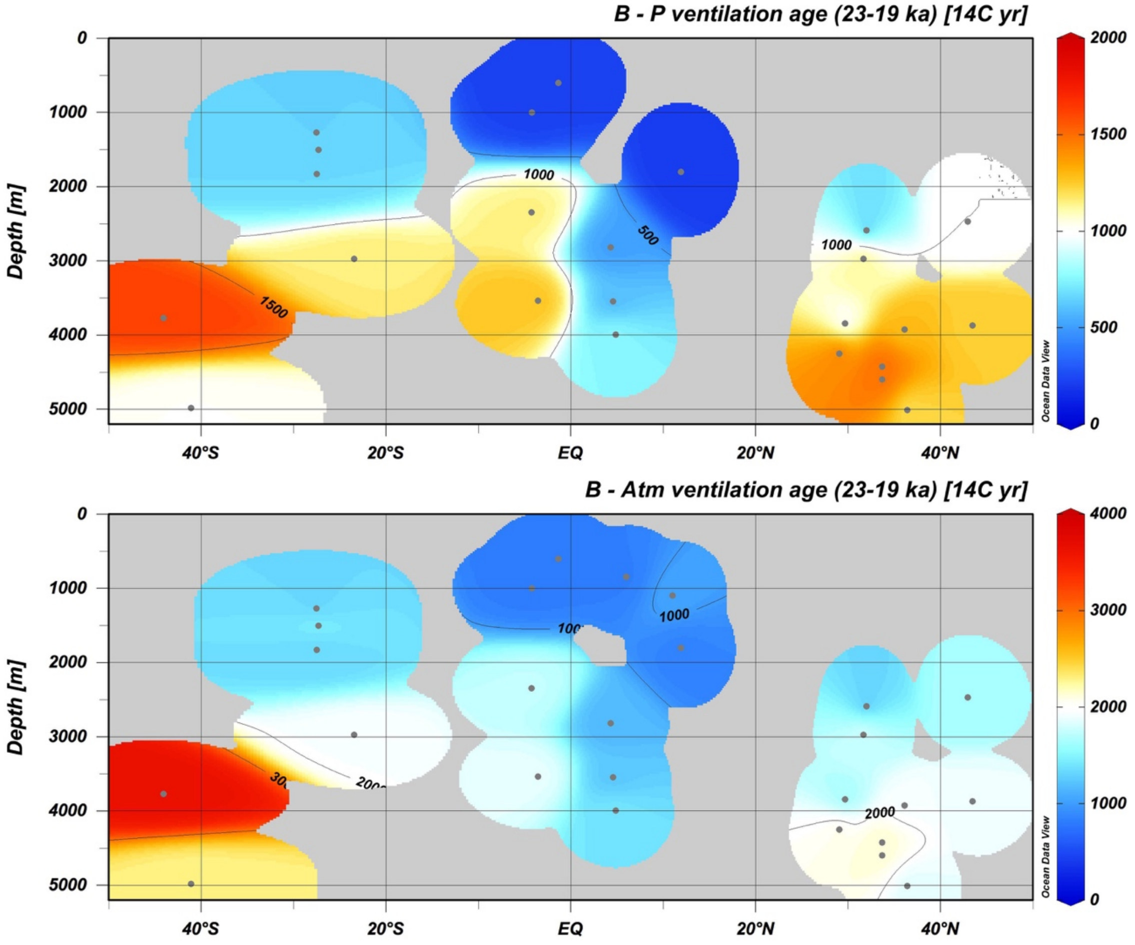

Supplementary Figure 6.

Compilation of benthic minus planktonic foraminifera (B-P) ^{14}C ages during 23-19 ka BP from the western Atlantic basin (upper panel). The B-P ventilation ages from the deep equatorial North Atlantic are younger than the values to the north and south, which might reflect bioturbation influences in those cores with low sedimentation rates[12]. The B-P ventilation age in the deep subtropical North Atlantic is similar to those in the deep Atlantic sector of the Southern Ocean, but the inferred surface reservoir age in the Southern Ocean is much larger[13,14], therefore leading to a larger B-Atm ventilation age in the deep Southern Ocean than the deep subtropical North Atlantic (lower panel; also including deep-sea coral data). Please note the difference between the scales of the two colour bars. See Methods and Supplementary Data 1 for the details of the compilation.

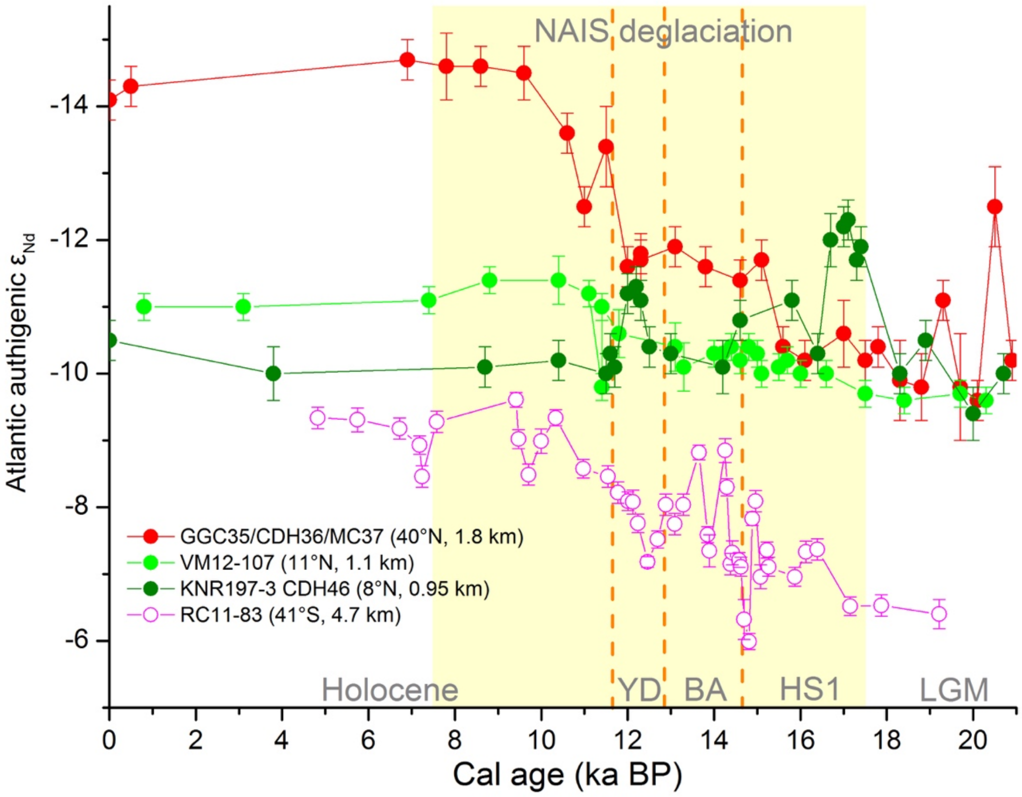

Supplementary Figure 7.

Evolution of authigenic ε_{Nd} from the intermediate tropical Atlantic (VM12-107 [15] and KNR197-3 CDH46 [16]) plotted against the records from the mid-depth North Atlantic (this study) and the deep South Atlantic[17]. Error bars plotted are 2σ. Site locations are shown in Supplementary Fig. 8.

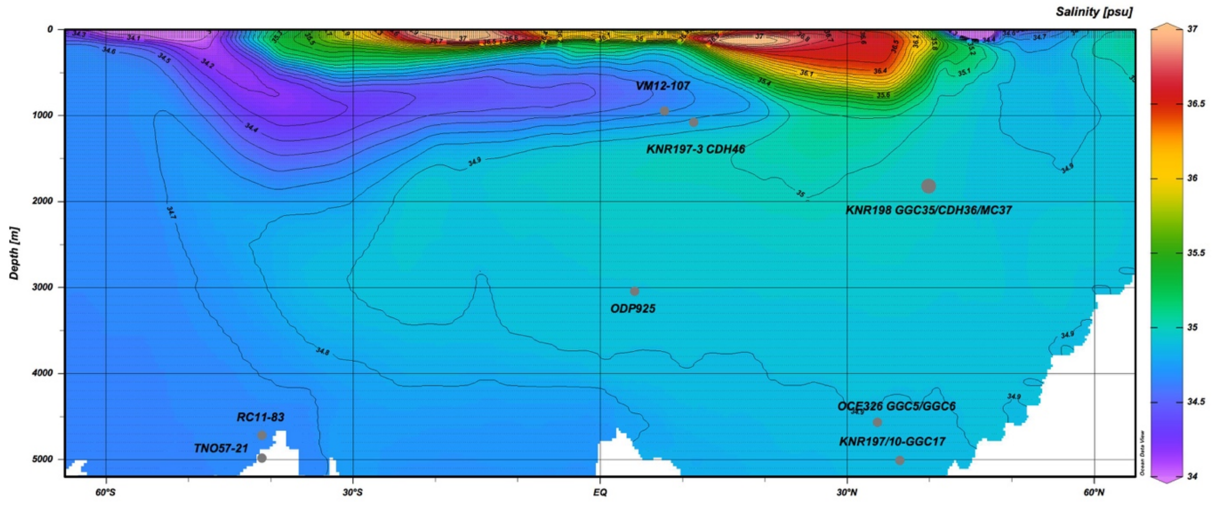

Supplementary Figure 8.

Locations of Atlantic ε_{Nd} records discussed in this study plotted on a modern salinity transect of the western Atlantic basin[18,19]. Large dot: core trio from this study; Other dots: cores in the deep North Atlantic [OCE326 GGC5/GGC6 (refs. [20,21]) and KNR197/10 GGC17 (ref. [22])], the deep South Atlantic [RC11-83 (ref. [17]) and TNO57-21 (refs. [23])], and the deep [ODP925 (refs. [24])] and intermediate [VM12-107 (ref. [15]) and KNR197-3 CDH46 (ref. [16])] equatorial Atlantic.

References

1 Piepgras, D. & Wasserburg, G. Rare earth element transport in the western North Atlantic inferred from Nd isotopic observations. *Geochim. Cosmochim. Acta* **51**, 1257-1271 (1987).

2 Lambelet, M. *et al.* Neodymium isotopic composition and concentration in the western North Atlantic Ocean: results from the GEOTRACES GA02 section. *Geochim. Cosmochim. Acta* **177**, 1-29 (2016).

3 Martin, E. E. *et al.* Extraction of Nd isotopes from bulk deep sea sediments for paleoceanographic studies on Cenozoic time scales. *Chem. Geol.* **269**, 414-431 (2010).

4 Taylor, S. & McLennan, S. *The continental crust: its composition and evolution*. 312 pp (Blackwell Press, 1985).

5 Gutjahr, M. *et al.* Reliable extraction of a deepwater trace metal isotope signal from Fe–Mn oxyhydroxide coatings of marine sediments. *Chem. Geol.* **242**, 351-370 (2007).

6 German, C. R. & Elderfield, H. Application of the Ce anomaly as a paleoredox indicator: the ground rules. *Paleoceanography* **5**, 823-833 (1990).

7 Stern, J. V. & Lisiecki, L. E. North Atlantic circulation and reservoir age changes over the past 41,000 years. *Geophys. Res. Lett.* **40**, 1-5 (2013).

8 van de Flierdt, T. *et al.* Neodymium in the oceans: a global database, a regional comparison and implications for palaeoceanographic research. *Phil. Trans. R. Soc. A* **374**, 20150293 (2016).

9 Marshall, J. & Schott, F. Open-ocean convection: Observations, theory, and models. *Rev. Geophys.* **37**, 1-64 (1999).

10 Pickart, R. S., Spall, M. A., Ribergaard, M. H., Moore, G. K. & Milliff, R. F. Deep convection in the Irminger Sea forced by the Greenland tip jet. *Nature* **424**, 152-156 (2003).

11 Labeyrie, L. D. *et al.* Changes in the vertical structure of the North Atlantic Ocean between glacial and modern times. *Quat. Sci. Rev.* **11**, 401-413 (1992).

12 Broecker, W. S., Peng, T.-H., Trumbore, S., bonani, G. & Wolfli, W. The distribution of radiocarbon in the glacial ocean. *Global Biogeochem. Cycles* **4**, 103-107 (1990).

13 Skinner, L. C., Fallon, S., Waelbroeck, C., Michel, E. & Barker, S. Ventilation of the deep Southern Ocean and deglacial CO_2 rise. *Science* **328**, 1147-1151 (2010).

14 Barker, S. & Diz, P. Timing of the descent into the last Ice Age determined by the bipolar seesaw. *Paleoceanography and Paleoclimatology* **29**, 489-507 (2014).

15 Xie, R. C., Marcantonio, F. & Schmidt, M. W. Reconstruction of intermediate water circulation in the tropical North Atlantic during the past 22,000 years. *Geochim. Cosmochim. Acta* **140**, 455-467 (2014).

16 Huang, K.-F., Oppo, D. W. & Curry, W. B. Decreased influence of Antarctic intermediate water in the tropical Atlantic during North Atlantic cold events. *Earth Planet. Sci. Lett.* **389**, 200-208 (2014).

17 Piotrowski, A. M., Goldstein, S. L., Hemming, S. R. & Fairbanks, R. G. Intensification and variability of ocean thermohaline circulation through the last deglaciation. *Earth Planet. Sci. Lett.* **225**, 205-220 (2004).

18 Schlitzer, R. Ocean Data View. *http://odv.awi.de* (2016).

19 Zweng, M. M. *et al.* World ocean atlas 2013. Volume 2, Salinity. (2013).

20 Roberts, N. L., Piotrowski, A. M., McManus, J. F. & Keigwin, L. D. Synchronous deglacial overturning and water mass source changes. *Science* **327**, 75-78 (2010).

21 McManus, J. F., Francois, R., Gherardi, J.-M., Keigwin, L. D. & Brown-Leger, S. Collapse and rapid resumption of Atlantic meridional circulation linked to deglacial climate changes. *Nature* **428**, 834-837 (2004).
22 Pöppelmeier, F., Gutjahr, M., Blaser, P., Keigwin, L. & Lippold, J. Origin of abyssal NW Atlantic water masses since the Last Glacial Maximum. *Paleoceanography and Paleoclimatology* **33**, 530-543 (2018).
23 Piotrowski, A. *et al.* Reconstructing deglacial North and South Atlantic deep water sourcing using foraminiferal Nd isotopes. *Earth Planet. Sci. Lett.* **357**, 289-297 (2012).
24 Howe, J. N., Piotrowski, A. M. & Rennie, V. C. Abyssal origin for the early Holocene pulse of unradiogenic neodymium isotopes in Atlantic seawater. *Geology* **44**, 831-834 (2016).
