# Supplementary material for: Glacial–interglacial Nd isotope variability of North Atlantic Deep Water modulated by North American ice sheet
Source: Nat Commun. 2019 Dec 18;10:5773. doi: 10.1038/s41467-019-13707-z (PMC6920363; doi:10.1038/s41467-019-13707-z)
Supplement: Supplementary file 2 — Description of Additional Supplementary Files [file 41467_2019_13707_MOESM2_ESM.pdf]

Description of Additional Supplementary Files

File Name: Supplementary Data 1
Description: Data generated or compiled during this study.
